# The incidence, risk factors and in-hospital mortality of acute kidney injury in patients after abdominal aortic aneurysm repair surgery

**DOI:** 10.1186/s12882-017-0594-6

**Published:** 2017-05-31

**Authors:** Ying Tang, Junzhe Chen, Kai Huang, Dan Luo, Peifen Liang, Min Feng, Wenxin Chai, Erik Fung, Hui Yao Lan, Anping Xu

**Affiliations:** 1 Department of Nephrology, Sun Yat-sen Memorial Hospital, Sun Yat-sen University, 107 Yan Jiang West Road, Guangzhou, China; 2 Department of Medicine and Therapeutics, Li Ka Shing Institute of Health Sciences, The Chinese University of Hong Kong, Shatin, Hong Kong; 3 Department of Nephrology, The People’s Hospital of Meishan City, Meishan, China; 4 Faculty of Medical Statistics and Epidemiology, School of Public Health, Sun Yat-sen University, Guangzhou, China

**Keywords:** Incidence, Risk factors, In-hospital mortality, Acute kidney injury, Abdominal aortic aneurysm

## Abstract

**Background:**

Acute kidney injury (AKI) is a severe complication associated with abdominal aortic aneurysm (AAA) repair. In this study, we evaluated the incidence, risk factors and in-hospital mortality of AKI in patients after the AAA repair surgery.

**Methods:**

A total of 314 Chinese AAA patients who underwent endovascular aneurysm repair (EVAR) or open aneurysm repair (OPEN) were enrolled in this study. AKI was diagnosed according to the 2012 KDIGO criteria. Logistic regression modeling was used to explore risk factors of AKI, while risk factors associated with in-hospital mortality in AKI patients were investigated using Cox proportional hazards model and Kaplan-Meier analysis, respectively. Multicollinearity analysis was performed to identify the collinearity between the variables before logistic regression analysis and Cox proportional hazards analysis.

**Results:**

Among 314 patients, 94 (29.9%) developed AKI after AAA repair surgery. Severity of AKI and ruptured AAA were independently associated with an increase in in-hospital mortality in AKI patients after AAA repair. Kaplan-Meier analysis identified severity of AKI as being negatively associated with hospital survival in AKI patients. Risk factors associated with AKI included cardiovascular disease (OR 3.169, 95% confidence interval (CI) 1.538 to 6.527, *P* = 0.002), decreased eGFR (OR 0.965, 95%CI 0.954 to 0.977, *P* < 0.001), ruptured AAA (OR 2.717, 95%CI 1.320 to 5.592, *P* = 0.007), renal artery involvement (OR 2.903, 95%CI 1.219 to 6.912, *P* = 0.016) and OPEN (OR 2.094, 95%CI 1.048 to 4.183, *P* = 0.036). Further subgroup analysis identified OPEN as an important risk factor of AKI in ruptured AAA patients but not in ruptured AAA patients. The incidence of AKI was significantly lower in EVAR than in OPEN (27.1% vs. 42.8%) and, similarly lower in nonruptured AAA than in ruptured AAA (26.2% vs. 48.1%).

**Conclusion:**

One-third of AAA patients developed AKI after repair surgery. Severity of AKI was associated with reduced survival rate in AAA patients who developed postoperative AKI. Decreased preoperative creatinine clearance, cardiovascular disease, ruptured AAA and OPEN were independent risk factors for postoperative AKI in all 314 AAA patients. Although a lower rate of incident AKI was observed in EVAR compared with OPEN, subgroup analysis of ruptured AAA versus nonruptured AAA showed that EVAR was an independent protective factor for AKI only in ruptured AAA patients but not in nonruptured AAA patients.

## Background

Abdominal aortic aneurysm (AAA) is a severe, devastating large artery disease that leads to a high mortality and morbidity, particularly when complicated by aortic rupture [1]. Surgery remains the first-line treatment for AAA, however, postoperative AKI is a major complication associated with poor outcomes [2]. Thus, identification of risk factors associated with postoperative AKI in AAA patients may improve the early monitoring, prevention and treatment of AKI after AAA repair surgery. Over the past decades, studies have reported the prevalence, risk factors and prognosis of postoperative AKI in AAA patients. However, the outcomes of these studies are inconsistent. The Risk, Injury, Failure, Loss of kidney function, and End-stage kidney disease (RIFLE) classification by the Acute Dialysis Quality Initiative group, or the Acute Kidney Injury Network (AKIN) criteria, has been used in most published studies, but results have been conflicting [3–5]. In 2012, the Kidney Disease Improving Global Outcomes (KDIGO) guideline writing group of the International Society of Nephrology proposed a new AKI criteria by combining AKIN, which has high sensitivity, with the well-stratified RIFLE [6]. However, little has been studied in the Chinese population, although a small sample size with critically ill patients after AAA repair has been reported previously [4]. To better evaluate the relationship between postoperative AKI and AAA repair surgery, we performed a retrospective analysis to define the incidence, risk factors and hospital mortality using the updated KDIGO criteria for classifying the severity of postoperative AKI.

## Methods

This was a retrospective study including two university hospitals of Sun Yat-sen University in Guangzhou, China: the First Affiliated Hospital and the Sun Yat-sen Memorial Hospital. Patients diagnosed as having AAA and who had undergone AAA surgery between November 1999 and December 2015 were included. The diagnosis of AAA was confirmed by computed tomography angiography (CTA). Exclusion criteria were 1) missing preoperative and postoperative serum creatinine (SCr) data within 7 days, and 2) end-stage renal disease requiring renal replacement therapy. The Institutional Review Boards of the hospitals approved this study and waived the need for informed consent. The study was conducted in accordance with the Declaration of Helsinki [7]. Patients’ medical records and laboratory results throughout the period were reviewed and demographic variables including age, sex and previous medical history (hypertension, diabetic mellitus, cardiovascular disease including coronary artery disease, heart failure and stroke) were recorded. Preoperative variables such as blood urea nitrogen (BUN), SCr and estimated glomerular filtration rate (eGFR) at admission, and the involvement of renal artery and AAA rupture were collected. In addition, the surgical approach, operation duration, blood pressure, volume of intraoperative hemorrhage and the need for vasoactive drugs during surgery were also recorded. Postoperative variables including serum levels of SCr and BUN at 24 h, 48 h and 7 days were collected.

AKI was defined according to the SCr-based criteria of KDIGO published in 2012. The diagnosis of AKI was defined as a SCr increase of ≥26.5 μmol/L within 48 h, or a 1.5-fold increase in SCr within 7 days above baseline value measured on admission. Data on urine output were not available for this study. Baseline eGFR was calculated using the CKD-EPI formula [8]. The severity of AKI was classified into three grades based on the postoperative peak SCr relative to baseline SCr according to the KDIGO criteria.

### Statistical analysis

Preoperative baseline characteristics, intraoperative and postoperative parameters of AAA patients and of subgroups were summarized by descriptive statistics. For continuous variables with symmetric distribution, mean data were expressed as mean ± standard deviations (SD), whereas median and interquartile range (IQR) was used for those with asymmetric distribution. Categorical variables were reported as number of patients with proportions. Student’s t-test, analysis of variance (ANOVA), chi-square, or Fisher’s exact test was used in univariate analyses of factors influencing the incidence of postoperative AKI. Risk factors found to be statistically significant from univariate analyses (*P* < 0.05) were then tested in multivariate analysis using logistic regression (Forward, LR) or Cox proportional hazards (Forward, LR) modeling. Logistic regression was used to test for risk factors associated with postoperative AKI. Cox proportional hazards model was used to identify risk factors associated with mortality. Multicollinearity analysis using Spearman’s correlation test (*r* > 0.5) was performed to identify the collinearity between the variables before logistic regression analysis and Cox proportional hazards analysis. Effect size estimate was represented by odds ratio (OR) with a 95% confidence interval (CI).Kaplan-Meier analysis and the log-rank test were used to compare survival between different postoperative AKI stages according to the KDIGO criteria. A *P*-value of <0.05 from two-sided tests was considered statistically significant. Analysis was performed using SPSS version 19 (IBM Corporation, Armonk, New York, USA).

## Results

### Preoperative baseline characteristics and intraoperative parameters of AAA patients

A total of 322 AAA patients diagnosed as having AAA and who had undergone AAA repair during the study period were analyzed. Among this study population, 8 patients were excluded from the study due to hemodialysis requirement (*n* = 3) and missing SCr values postoperatively (*n* = 5). Details of preoperative baseline characteristics, intraoperative and postoperative parameters were shown in Table 1. In 314 AAA patients, 263 (83.8%) were male. The median age was 69 years (IQR 63 to 75). The mean baseline eGFR on admission was 69.3 ml/min/1.73m^2^ and the median baseline SCr was 90.0(IQR, 75.8 to 114.2) μmol/L. Thirty patients (9.6%) with AAA had renal artery involvement. Fifty-four of 314 (17.2%) patients had ruptured AAA. Hypertension, diabetes mellitus and a history of cardiovascular disease were present in 186 (59.2%), 26 (8.3%) and 48 (15.3%) patients, respectively. Two hundred fifty-eight patients (82.2%) were treated with EVAR and 56 patients (17.8%) had undergone OPEN. The median operation time for all AAA patients was 230.0 (IQR, 180.0 to 285.0) min and 18.2% patients had intraoperative hemorrhage > 1 L.

**Table 1:** Preoperative baseline characteristics, intraoperative and postoperative parameters of AAA patients

Parameter	All (*n* = 314)	Surgical procedures			Rupture status		
		Endovascular aortic repair (*n* = 258)	Open aneurysm repair (*n* = 56)	*P*	Ruptured AAA (*n* = 54)	Non-ruptured AAA (*n* = 260)	*P*
Age (years)	69.0 (63.0–75.0)	69.0 (63.0–75.0)	66.5 (59.3–73.0)	0.157	68.5 (60.8–75.0)	69.0 (64.0–74.8)	0.471
Male sex (n, %)	263 (83.8%)	218 (84.5%)	45 (80.4%)	0.447	47 (87.0%)	216 (83.1%)	0.473
Baseline SCr (umol/L)	90.0 (75.8–114.2)	89.0 (75.0–112.0)	99.5 (78.0–128.8)	0.241	105.0 (75.8–172.8)	90.0 (75.3–108.0)	0.015
Baseline blood urea nitrogen (mmol/L)	5.9 (4.7–7.7)	5.8 (4.7–7.6)	6.1 (4.8–7.9)	0.502	7.4 (4.8–10.9)	5.8 (4.7–7.3)	0.006
Baseline eGFR (ml/min/1.73m^2^)	69.3 ± 25.4	69.3 ± 24.5	69.4 ± 29.6	0.982	61.5 ± 31.5	71.0 ± 23.7	0.040
Ruptured abdominal aortic aneurysm (n, %)	54 (17.2%)	41 (15.9%)	13 (23.2%)	0.188	—	—	—
Renal artery involvement (n, %)	30 (9.6%)	22 (8.5%)	8 (14.3%)	0.184	0 (0.0%)	30 (11.5%)	0.004
Hypertension (n, %)	186 (59.2%)	153 (59.3%)	33 (58.9%)	0.959	33 (61.1%)	153 (58.8%)	0.758
Cardiovascular diseases (n, %)	48 (15.3%)	43 (16.7%)	5 (8.9%)	0.145	4 (7.4%)	44 (16.9%)	0.096
Diabetes mellitus (n, %)	26 (8.3%)	22 (8.5%)	4 (7.1%)	0.733	5 (9.3%)	21 (8.1%)	0.774
Surgical procedures							
Open aneurysm repair (n, %)	56 (17.8%)	—	—	—	13 (24.1%)	43 (16.5%)	0.188
Endovascular aneurysm repair (n, %)	258 (82.2%)	—	—	—	41 (75.9%)	217 (83.5%)	0.188
Operative duration > 230 min (n, %)	150 (47.8%)	114 (44.2%)	36 (64.3%)	0.006	32 (59.3%)	118 (45.4%)	0.063
Intraoperative hemorrhage > 1 L (n, %)	57 (18.2%)	38 (14.7%)	19 (33.9%)	0.001	29 (53.7%)	28 (10.8%)	<0.001
Vasoactive support (n, %)	38 (11.8%)	25 (9.7%)	13 (23.2%)	0.005	26 (48.1%)	12 (4.6%)	<0.001
AKI (n, %)	94 (29.9%)	70 (27.1%)	24 (42.8%)	0.020	26 (48.1%)	68 (26.2%)	0.001

### Incidence and risk factors associated with AKI in AAA patients after repair surgery

According to medical records, the incidence of AKI following AAA repair diagnosed by surgeons at discharge was 2.5% (*n* = 8), whereas our data showed that the incidence of AKI defined by 2012 KDIGO criteria was 29.9% (*n* = 94), a 12-fold difference. Using the KDIGO criteria, we observed that 65 patients were in Stage 1(69.1%), 17 in Stage 2(18.1%), and 12 in Stage 3(12.8%) AKI. We found that the independent variables, “rupture” and “intraoperative hemorrhage”, and “vasoactive support” were highly correlated as indicated by Spearman’s correlation test (*r* > 0.5). Moreover, there was significant collinearity between these variables. Based on these findings, we excluded intraoperative hemorrhage and vasoactive drug support in the subsequent logistic regression analysis of AKI in all AAA patients, in subgroup analysis of operative method, and in Cox proportional hazards analysis. Compared with non-AKI patients after AAA repair, decreased preoperative baseline eGFR, cardiovascular disease history, ruptured AAA and OPEN were risk factors associated with postoperative AKI in all 314 AAA patients (Table 2).

**Table 2:** Risk factors of postoperatively AKI following AAA repair surgery

Parameter			Logistic analysis			
			Univariate analysis		Multivariate analysis	
	AKI (*n* = 94)	Non-AKI (*n* = 220)	OR (95%CI)	*P*	OR (95%CI)	*P*
Age (years)	70.5 (64.0–77.3)	68.0 (62.0–73.0)	1.035 (1.008–1.063)	0.010		
Male sex (%)	84 (89.4%)	179 (81.4%)	1.924 (0.920–4.026)	0.082		
Baseline SCr (umol/L)	113.0 (90.0–157.5)	85.0 (72.3–101.0)	1.006 (1.002–1.010)	0.007		
Baseline blood urea nitrogen (mmol/L)	7.25 (5.28–10.33)	5.6 (4.5–7.0)	1.280 (1.167–1.405)	<0.001		
Baseline eGFR (ml/min/1.73m^2^)	53.7 ± *23.6*	76.0 ± *23.2*	0.960 (0.948–0.972)	<0.001	0.965 (0.954–0.977)	<0.001
Ruptured abdominal aortic aneurysm (n, %)	*2*6 (27.7%)	*2*8 (12.7%)	2.622 (1.437–4.783)	0.002	2.717 (1.320–5.592)	0.007
Renal artery involvement (%)	14 (14.9%)	16 (7.3%)	2.231 (1.041–4.783)	0.039	2.903 (1.219–6.912)	0.016
Hypertension (%)	70 (74.5%)	116 (52.7%)	2.615 (1.533–4.460)	<0.001		
Cardiovascular diseases (%)	25 (26.6%)	23 (10.5%)	3.103 (1.654–5.822)	<0.001	3.169(1.538-6.527)	0.002
Diabetes mellitus (%)	11 (11.7%)	15 (6.8%)	1.811 (0.799–4.107)	0.155		
Surgical procedures						
EVAR (%, reference)	70 (74.5%)	188 (85.5%)	1		1	
OPEN (%)	24 (25.5%)	32 (14.5%)	2.014 (1.110–3.656)	0.021	2.094 (1.048–4.183)	0.036
Operative duration > 230 min (n, %)	57 (60.6%)	93 (42.3%)	1.975 (1.209–3.228)	0.007		

*EVAR* endovascular aortic repair, *OPEN* open aneurysm repair surgery

Comparing 258 patients who received EVAR and 56 in OPEN, there was no significant difference in preoperative baseline parameters, including age, gender, baseline BUN, SCr and eGFR, renal artery involvement, ratio of ruptured AAA, and history of hypertension, cardiovascular disease and diabetes (Table 1). Compared with OPEN, EVAR was associated with shorter operative time, less intraoperative hemorrhage, and less vasoactive drug support (Table 1). Furthermore, a lower incidence of AKI was associated with EVAR (27.1%) than with OPEN (42.8%) (Table 1). In order to separately analyze the risk factors for AKI in EVAR and OPEN, we grouped AKI patients according to surgical method. In the EVAR subgroup, multivariate analysis identified an association between postoperative AKI and decreased preoperative baseline eGFR, renal artery involvement, and a history of cardiovascular disease (Table 3). In the OPEN subgroup, decreased preoperative baseline eGFR and ruptured AAA were associated with postoperative AKI (Table 3).

**Table 3:** Comparison of risk factors for postoperatively AKI in AAA patients undergoing endovascular aortic repair and open aneurysm repair

Parameter	EVAR				OPEN			
	Univariate analysis		Multivariate analysis		Univariate analysis		Multivariate analysis	
	OR (95%CI)	*P*	OR (95%CI)	*P*	OR (95%CI)	*P*	OR (95%CI)	*P*
Age (years)	1.033 (1.001–1.066)	0.044			1.053 (1.000–1.110)	0.050		
Male sex (%)	1.338 (0.602–2.975)	0.475			10.455 (1.234–88.601)	0.031		
Baseline SCr (umol/L)	1.003 (1.000–1.007)	0.065			1.065 (1.029–1.102)	<0.001		
Baseline blood urea nitrogen (mmol/L)	1.223 (1.114–1.343)	<0.001			1.862 (1.305–2.656)	0.001		
Baseline eGFR (ml/min/1.73m^2^)	0.966 (0.953–0.978)	<0.001	0.968 (0.956–0.981)	<0.001	0.925 (0.887–0.964)	<0.001	0.918 (0.872–0.966)	0.001
Ruptured abdominal aortic aneurysm (n,%)	1.491 (0.730–3.043)	0.273			31.000 (3.625–265.087)	0.002	27.052 (2.132–343.324)	0.011
Renal artery involvement (%)	3.000 (1.237–7.277)	0.015	3.120 (1.200–8.107)	0.020	0.771 (0.165–3.601)	0.741		
Hypertension (%)	2.263 (1.242–4.123)	0.008			4.886 (1.460–16.345)	0.010		
Cardiovascular diseases (%)	2.870 (1.457–5.651)	0.002	2.324 (1.104–4.891)	0.026	—————			
Diabetes mellitus (%)	1.604 (0.642–4.007)	0.312			4.429 (0.431–45.516)	0.211		
Operative duration > 230 min (n, %)	1.616 (0.931–2.807)	0.088			3.353 (1.005–11.190)	0.049		

Note: History of cardiovascular disease and diabetes mellitus were not recorded in the non-AKI group in Open aneurysm repair AAA patients, so logistic analysis was not possible.

*EVAR* endovascular aortic repair, *OPEN* open aneurysm repair surgery

Considering the different clinical situations that may exist between ruptured and nonruptured AAA patients, we also grouped AKI patients according to whether they had ruptured or nonruptured AAA, and compared their baseline parameters and risk factors associated with postoperative AKI. As shown in Table 1, the incidence of AKI occurring with ruptured AAA (48.1%) was higher than with nonruptured AAA (26.2%). Ruptured AAA patients had higher baseline BUN and SCr, lower baseline eGFR, more renal artery involvement, higher incidence of blood loss > 1 L and more vasoactive drug support compared with nonruptured AAA patients (Table 1). There was no statistical difference in age, sex, history of hypertension, history of cardiovascular disease and diabetes, or surgical repair method used between ruptured and nonruptured groups (Table 1). Risk factors associated with postoperative AKI in patients with ruptured AAA included decreased baseline eGFR and OPEN, whereas in nonruptured AAA patients with postoperative AKI, risk factors included decreased baseline eGFR, renal artery involvement, a history of cardiovascular disease, and intraoperative hemorrhage (Table 4).

**Table 4:** Comparison of risk factors for postoperatively AKI in patients with ruptured and nonruptured AAA

Parameter	Ruptured AAA				Nonruptured AAA			
	Univariate analysis		Multivariate analysis		Univariate analysis		Multivariate analysis	
	OR (95%CI)	*P*	OR (95%CI)	*P*	OR (95%CI)	*P*	OR (95%CI)	*P*
Age (years)	1.017 (0.973–1.064)	0.448			1.050 (1.015–1.087)	0.005		
Male sex (%)	2.609 (0.459–14.814)	0.279			1.731 (0.761–3.937)	0.191		
Baseline SCr (umol/L)	1.010 (1.001–1.019)	0.033			1.006 (0.009–1.007)	0.132		
Baseline blood urea nitrogen (mmol/L)	1.259 (1.061–1.495)	0.008			1.238 (1.112–1.377)	<0.001		
Baseline eGFR (ml/min/1.73m^2^)	0.973 (0.953–0.992)	0.007	0.973 (0.951–0.994)	0.014	0.956 (0.942–0.971)	<0.001	0.957 (0.942–0.973)	<0.001
Renal artery involvement (%)	——————				2.852 (1.308–6.217)	0.008	2.733 (1.112–6.718)	0.029
Hypertension (%)	1.950 (0.639–5.952)	0.241			2.928 (1.563–5.486)	0.001		
Cardiovascular diseases (%)	——————				3.283 (1.673–6.442)	0.001	2.567 (1.167–5.647)	0.019
Diabetes mellitus (%)	——————				1.142 (0.424–3.073)	0.793		
Surgical procedures								
EVAR (%, reference)	1		1		1			
OPEN (%)	23.143 (2.724–196.639)	0.004	23.385 (2.564–213.280)	0.005	1.113 (0.535–2.315)	0.775		
Operative duration > 230 min (n, %)	0.882 (0.298–2.615)	0.821			2.268 (1.289–3.990)	0.005		
Intraoperative hemorrhage > 1 L (n, %)	3.477 (1.127–10.726)	0.030			3.897 (1.745–8.703)	0.001	6.053 (2.384–15.372)	<0.001

Note: Factors including renal artery involvement, history of cardiovascular disease and diabetes mellitus were not recorded in the AKI group in ruptured AAA patients, so logistic analysis was not suitable.

*EVAR* endovascular aortic repair, *OPEN* open aneurysm repair surgery

### In-hospital mortality of AKI in AAA patients after repair surgery

**Fig. 1:**
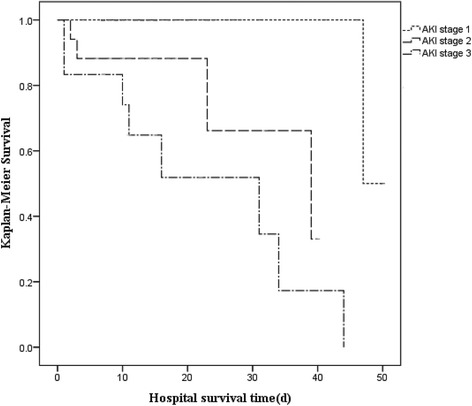
Kaplan-Meier analysis of hospital survival in 92 postoperatively AKI AAA patients according to AKI stage (log-rank test: *P* < 0.001)

Overall in-hospital mortality of AKI patients (13.8%) was 4.3-fold higher than that of non-AKI patients (3.2%). Cox proportional hazards analysis for in-hospital mortality of AKI patients was presented in Table 5. Univariate analysis revealed that ruptured AAA, higher level of peak SCr after repair surgery, and more advanced AKI Stage were risk factors associated with increased in-hospital mortality. Multivariate analysis identified ruptured AAA and severity of AKI to be associated with increased in-hospital mortality. Kaplan-Meier analysis showed that improved survival rate was inversely related to the severity of AKI (Fig. 1).

**Table 5:** Analysis of risk factors predictive of mortality in AKI patients undergoing AAA repair surgery

Parameter	Univariate analysis		Multivariate analysis	
	HR (95%CI)	*P*	HR (95%CI)	*P*
Age (years)	0.978 (0.917–1.042)	0.488		
Male sex (%)	23.431 (0.000–259.795)	0.595		
Baseline SCr (umol/L)	0.999 (0.990–1.008)	0.868		
Baseline blood urea nitrogen (mmol/L)	0.940 (0.802–1.101)	0.442		
Baseline eGFR (ml/min/1.73m^2^)	1.003 (0.977–1.029)	0.848		
Ruptured abdominal aortic aneurysm (%)	3.637 (1.174–11.271)	0.025	6.814 (1.344–34.543)	0.020
Renal artery involvement (%)	0.939 (0.239–3.700)	0.929		
Hypertension (%)	0.889 (0.182–4.346)	0.884		
Cardiovascular diseases (%)	0.869 (0.238–3.174)	0.832		
Diabetes mellitus (%)	0.480 (0.061–3.766)	0.485		
Surgical procedures				
EVAR (%, reference)	1			
Open aneurysm repair (%)	2.062 (0.663–6.410)	0.211		
Operative duration > 230 min (n, %)	0.962 (0.278–3.222)	0.950		
Peak SCr after surgery (umol/L)	1.003 (1.000–1.005)	0.023		
Peak blood urea nitrogen after surgery (mmol/L)	1.028 (0.986–1.071)	0.192		
AKI stage		0.001		0.006
Stage 1(reference)	1		1	
Stage 2	11.973 (1.326–108.090)	0.027	12.856 (1.279–129.186)	0.030
Stage 3	41.532 (5.029–342.998)	0.001	37.649 (3.766–376.362)	0.002
Duration to AKI diagnosis (d)	0.133 (0.009–2.058)	0.149		
Duration to peak SCr (d)	1.046 (0.903–1.211)	0.551

## Discussion

Our results showed that the incidence of postoperative AKI was 29.9%, and multivariate analysis identified a history of cardiovascular disease, decreased baseline eGFR, involvement of renal artery, ruptured AAA, and OPEN as risk factors of postoperative AKI in all 314 AAA patients undergoing repair surgery. Patients with AKI were at risk for increased in-hospital mortality when compared to non-AKI patients. Moreover, the severity of AKI and ruptured AAA were associated with increased in-hospital mortality in patients with postoperative AKI. Kaplan-Meier analysis showed that improved survival rate was inversely correlated with the severity of AKI in postoperative AKI patients. Importantly, the incidence of AKI was significantly lower in EVAR compared with OPEN. Although OPEN was associated with postoperative AKI, further subgroup analysis found that this increased risk was primarily limited to ruptured, and not nonruptured, AAA patients. Collectively, these findings suggest that EVAR may be a safer approach to AAA repair surgery than OPEN for the prevention of postoperative AKI, especially for patients with ruptured AAA.

In this study, the incidence of postoperative AKI was found to be 29.9%, considerably higher than that reported in the literature [5, 9]. This may be associated with the use of different diagnostic criteria for AKI between this and other studies in which the AKIN or RIFLE criteria were applied and about 9% of AKI patients at the early stage may have been missed by the RIFLE criteria, and 26.9% of AKI patients detected by RIFLE were missed by AKIN [10, 11]. Our results suggested that the KDIGO criteria may be used preoperatively to assess for the risk of postoperative AKI in AAA patients, and may improve early identification and possibly preemptive management of AKI. Unexpectedly, we found that the clinical diagnosis of AKI at the time of hospital discharge was only 2.5%, indicating that AKI may have been markedly underestimated clinically when early diagnosis of AKI was missed.

AKI is an important risk factor associated with long-term mortality of AAA patients [2, 5]. In this study we also found higher in-hospital mortality in AKI patients compared with non-AKI patients after AAA repair surgery (13.8% vs. 3.2%, *P* < 0.001). In addition, more severe AKI was associated with higher in-hospital mortality and lower survival rate in patients with postoperative AKI. Consistent with previous studies [4, 12–15], a history of cardiovascular disease, decreased baseline eGFR, renal artery involvement, ruptured AAA, and OPEN were risk factors for postoperative AKI.

While there is a growing body of evidence supporting the use of EVAR as a less invasive approach to achieving better clinical outcomes including a reduction in postoperative AKI compared with OPEN in patients undergoing AAA repair [16, 17], the superiority of EVAR over OPEN remainscontroversial [18–22]. One study even found that more severe renal impairment occurred in the EVAR compared with OPEN, which may be attributable to atherosclerotic embolization or contrast-induced nephropathy [23]. In this study, we observed a lower incidence of postoperative AKI in AAA patients treated by EVAR when compared with OPEN. This may be associated with less intraoperative hemorrhage, shorter operation time, and less vasoactive drug support in patients treated with EVAR. Although multivariate analysis identified that OPEN was associated with postoperative AKI in all 314 AAA patients (*P* = 0.036), distinct results were drawn from ruptured or nonruptured subgroup analysis. OPEN was associated with postoperative AKI (OR 23.385, CI 2.564–213.280, *P* = 0.005) in ruptured but not nonruptured AAA patients. This phenomenon may be partly explained by mild or moderate disease characterized by less intraoperative hemorrhage in nonruptured patients which could not exploit the major advantage of EVAR over OPEN. Thus, EVAR appears to be superior to OPEN in this study in terms of incidence of postoperative AKI for ruptured AAA patients in the prevention of postoperative AKI.

This study has some limitations inherent in a retrospective analysis. First, selection bias, including an unequal number of patients receiving EVAR versus OPEN, a large majority of study participants with mild AKI, and nonrandomized distribution of patients, is inevitable. Second, this study did not collect information on the quantity and type of iodinated contrast agents used in EVAR, and thus, the effects of contrast agents on postoperative AKI in EVAR could not be analyzed. Contrast-induced nephropathy is a serious complication that impacts on survival and recently, even questions about the safety of, and reduced harm from, hypoosmolar iodinated contrast agents have been raised. Post-EVAR AKI might not be directly related to contrast medium but might predominantly be influenced by other factors [24]. Moreover, some studies have demonstrated that iodinated contrast volume is not an independent risk factor for AKI [12, 25]. With the advent of less nephrotoxic iodinated contrast, we believe that the injury effect of iodinated contrast on kidney will gradually become less important. Third, this study had a relatively short period of follow up, which only tracked short-term outcomes. Long-term outcomes of postoperative AKI in AAA patients in this study remain to be documented. Lastly, all 314 patients were not randomly assigned to receive EVAR or OPEN, although the baseline parameters of patients in these two groups were similar. To reduce potential bias caused by these mixed factors, we adopted methods including subgroup analysis to acquire more credible results. In subgroup analysis according to the status of the AAA (ruptured or nonruptured), we demonstrated that OPEN was an important risk factor associated with postoperative AKI in ruptured AAA patients but not in nonruptured AAA patients. A randomized, prospective study in the Chinese population is awaited to confirm whether EVAR is superior to OPEN in minimizing postoperative AKI associated with AAA repair surgery.

## Conclusion

This study demonstrates that one-third of AAA patients can develop AKI after aneurysm repair surgery and that the severity of AKI and ruptured AAA status are associated with increased in-hospital mortality in AAA patients who develop postoperative AKI. The incidence of AKI is significantly lower in EVAR compared with OPEN, and OPEN is a risk factor for postoperative AKI particularly in patients with ruptured AAA.

## Abbreviations

AAA: Abdominal aortic aneurysm
AKI: Acute kidney injury
AKIN: Acute Kidney Injury Network
BUN: Blood urea nitrogen
eGFR: Estimated glomerular filtration rate
EVAP: Endovascular aneurysm repair
KDIGO: Kidney Disease: Improving Global Outcomes
OPEN: Open aneurysm repair
RIFLE: The Risk, Injury, Failure, Loss of kidney function, and End-stage kidney disease
SCr: Serum creatinine

## References

[1] Setacci F, Galzerano G, De Donato G, Benevento D, Guerrieri MW (2016). Abdominal aortic aneurysm. J Cardiovasc Surg.

[2] Kopolovic I, Simmonds K, Duggan S, Ewanchuk M, Stollery DE (2013). Risk factors and outcomes associated with acute kidney injury following ruptured abdominal aortic aneurysm. BMC Nephrol.

[3] van Beek SC, Legemate DA, Vahl A, Bouman CS, Vogt L (2014). Acute kidney injury defined according to the ‘Risk’, ‘Injury’, ‘Failure’, ‘Loss’ and ‘End-stage’ (RIFLE) criteria after repair for a ruptured abdominal aortic aneurysm. J Vasc Surg.

[4] Yue JN, Luo Z, Guo DQ, Xu X, Chen B (2013). Evaluation of acute kidney injury as defined by the risk, injury, failure, loss, and end-stage criteria in critically ill patients undergoing abdominal aortic aneurysm repair. Chin Med J.

[5] Saratzis A, Melas N, Mahmood A, Sarafidis P (2015). Incidence of acute kidney injury (AKI) after endovascular abdominal aortic aneurysm repair (EVAR) and impact on outcome. Eur J Vasc Endovasc Surg.

[6] Kellum JA, Lameire N, Group KAGW (2013). Diagnosis, evaluation, and management of acute kidney injury: a KDIGO summary (part 1). Crit Care.

[7] World Medical Association Declaration of Helsinki: Ethical Principles for Medical Research Involving Human Subjects. J Am Med Asso. 2013;310(20):2191–94. 10.1001/jama.2013.281053 24141714

[8] Flamant M, Haymann JP, Vidal-Petiot E, Letavernier E, Clerici C (2012). GFR estimation using the Cockcroft-Gault, MDRD study, and CKD-EPI equations in the elderly. Am J Kidney Dis.

[9] Bang JY, Lee JB, Yoon Y, Seo HS, Song JG (2014). Acute kidney injury after infrarenal abdominal aortic aneurysm surgery: a comparison of AKIN and RIFLE criteria for risk prediction. Br J Anaesth.

[10] Joannidis M, Metnitz B, Bauer P, Schusterschitz N, Moreno R (2009). Acute kidney injury in critically ill patients classified by AKIN versus RIFLE using the SAPS 3 database. Intensive Care Med.

[11] Mizuno T, Sato W, Ishikawa K, Shinjo H, Miyagawa Y (2012). KDIGO (Kidney Disease: Improving Global Outcomes) criteria could be a useful outcome predictor of cisplatin-induced acute kidney injury. Oncology.

[12] Ambler GK, Coughlin PA, Hayes PD, Varty K, Gohel MS (2015). Incidence and outcomes of severe renal impairment following ruptured abdominal aortic aneurysm repair. Eur J Vasc Endovasc Surg.

[13] Saratzis A, Nduwayo S, Sarafidis P, Sayers RD, Bown MJ (2016). Renal function is the main predictor of acute kidney injury after endovascular abdominal aortic aneurysm repair. Ann Vasc Surg.

[14] Kamitani K, Yoshida H, Arai R, Ito H, Miyoshi H (2011). Examination of acute kidney injury after abdominal aortic aneurysm surgery. Masui.

[15] Kim M, Brady JE, Li G (2014). Anesthetic technique and acute kidney injury in endovascular abdominal aortic aneurysm repair. J Cardiothorac Vasc Anesth.

[16] Greenhalgh RM, Brown LC, Kwong GP, Powell JT, Thompson SG (2004). Comparison of endovascular aneurysm repair with open repair in patients with abdominal aortic aneurysm (EVAR trial 1), 30-day operative mortality results: randomised controlled trial. Lancet.

[17] Lovegrove RE, Javid M, Magee TR, Galland RB (2008). A meta-analysis of 21, 178 patients undergoing open or endovascular repair of abdominal aortic aneurysm. Br J Surg.

[18] Hua HT, Cambria RP, Chuang SK, Stoner MC, Kwolek CJ (2005). Early outcomes of endovascular versus open abdominal aortic aneurysm repair in the National Surgical Quality Improvement Program-Private Sector (NSQIP-PS). J Vasc Surg.

[19] Prinssen M, Verhoeven EL, Buth J, Cuypers PW, van Sambeek MR (2004). A randomized trial comparing conventional and endovascular repair of abdominal aortic aneurysms. N Engl J Med.

[20] Antoniou GA, Georgiadis GS, Antoniou SA, Pavlidis P, Maras D (2013). Endovascular repair for ruptured abdominal aortic aneurysm confers an early survival benefit over open repair. J Vasc Surg.

[21] Aziz F, Azab A, Schaefer E, Reed AB (2016). Endovascular repair of ruptured abdominal aortic aneurysm is associated with lower incidence of post-operative acute renal failure. Ann Vasc Surg.

[22] Pirgakis KM, Makris K, Dalainas I, Lazaris AM, Maltezos CK (2014). Urinary cystatin C as an early biomarker of acute kidney injury after open and endovascular abdominal aortic aneurysm repair. Ann Vasc Surg.

[23] Hinchliffe RJ, Bruijstens L, Mac Sweeney ST, Braithwaite BD (2006). A randomised trial of endovascular and open surgery for ruptured abdominal aortic aneurysm - results of a pilot study and lessons learned for future studies. Eur J Vasc Endovasc Surg.

[24] Davenport MS, Cohan RH, Khalatbari S, Ellis JH (2014). The challenges in assessing contrast-induced nephropathy: where are we now?. Am J Roentgenol.

[25] Sailer AM, Nelemans PJ, van Berlo C, Yazar O, de Haan MW, Fleischmann D, Schurink GW (2016). Endovascular treatment of complex aortic aneurysms: prevalence of acute kidney injury and effect on long-term renal function. Eur Radiol.

